# Hydraulic Fracture Extending into Network in Shale: Reviewing Influence Factors and Their Mechanism

**DOI:** 10.1155/2014/847107

**Published:** 2014-06-15

**Authors:** Lan Ren, Jinzhou Zhao, Yongquan Hu

**Affiliations:** State Key Laboratory of Oil and Gas Reservoir Geology & Exploitation, Southwest Petroleum University, Chengdu 610500, China

## Abstract

Hydraulic fracture in shale reservoir presents complex network propagation, which has essential difference with traditional plane biwing fracture at forming mechanism. Based on the research results of experiments, field fracturing practice, theory analysis, and numerical simulation, the influence factors and their mechanism of hydraulic fracture extending into network in shale have been systematically analyzed and discussed. Research results show that the fracture propagation in shale reservoir is influenced by the geological and the engineering factors, which includes rock mineral composition, rock mechanical properties, horizontal stress field, natural fractures, treating net pressure, fracturing fluid viscosity, and fracturing scale. This study has important theoretical value and practical significance to understand fracture network propagation mechanism in shale reservoir and contributes to improving the science and efficiency of shale reservoir fracturing design.

## 1. Introduction

Hydraulic fracturing has become an important technique to improve well production and the recovery of low-permeability reservoirs in the oil and gas field development. In recent years, especially, under the impetus of the multistage fracturing technique of horizontal well [1–3], a great success has been achieved in developing unconventional natural gas of low permeability shale reservoirs in USA, and its annual production in 2011 reached 1720 × 10^8^ m^3^, far more than 2011 natural gas total production in China 1025.3 × 10^8^ m^3^.

For a long time, hydraulic fracture geometry in the far well zone is one of the latest leading-edge theories in hydraulic fracturing. According to the traditional classical fracturing theory, hydraulic fracture in the far well zone is a symmetry plane biwing fracture extending in the direction perpendicular to the minimum horizontal principal stress. In recent years, many researchers have recognized the existence of the complex hydraulic fracture extension. Warpinski et al. [4–6] discovered the phenomenon that the main fracture and the branch fractures extended simultaneously through the field tests and put forward the concept of fracture propagation zone. Afterwards, through physical simulation experiments, Blanton [7, 8] and Chen et al. [9–12] found that hydraulic fracture presented three kinds of extension path when it intersected with natural fractures: crossing natural fractures, extending along natural fractures, or the two cases occurring simultaneously. Mahrer [13] considered fracture network would form during fracturing process of naturally fractured formations. Beugelsdijk et al. [14] confirmed the existence of the fracture network and found that the fracture network would easily form under low fluid viscosity injection through laboratory experiments. Fisher and Maxwell [15–18] found that hydraulic fracture would extend into network during fracturing of the shale reservoir through microseismic monitoring mapping. Mayerhofer et al. [19, 20] proposed that good stimulation effect of shale reservoirs could be achieved by increasing the stimulated reservoir volume (SRV) to form a maximum area of the fracture network, and the stimulation success of shale reservoirs depended on whether hydraulic fracture could extend into the fracture network. Due to the insufficient and imperfect understanding of the fracture network forming mechanism in shale reservoirs, there is always blindness in the fracturing design of shale reservoir. Based on the research results of laboratory experiments, field fracturing practices, theoretical analysis, and numerical simulation, this paper analyzes the factors that influence hydraulic fracture propagation in shale reservoirs, which has an important theoretical significance to improve the reliability of the fracturing design for shale reservoirs.

## 2. Hydraulic Fractures Extending Characteristic in Shale

**Figure 1:**
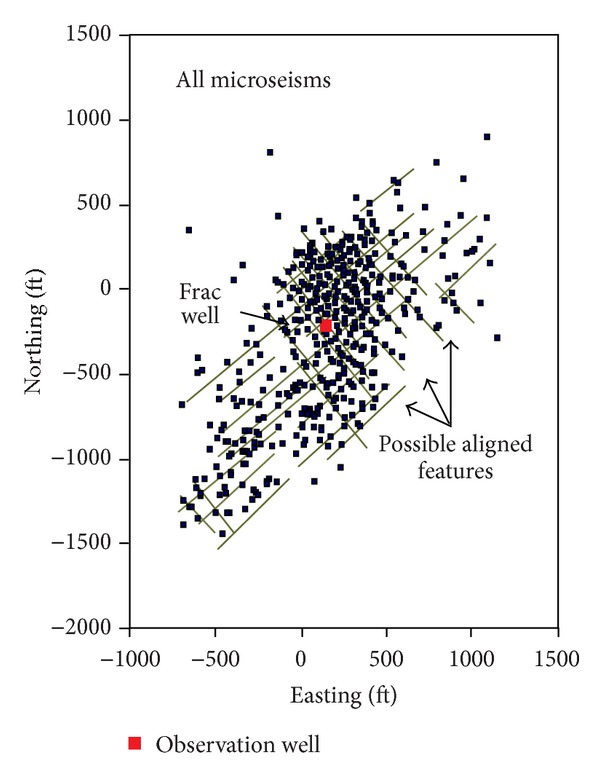
Microseismic mapping confirmed complex fracture extension in shale reservoirs [21].

Microseismic mapping showed that hydraulic fracture in shale was a complex fracture network system which consisted of multiple irregular fractures [21] as shown in Figure 1. The Barnett shale natural fractures' direction was the north by west, and the propagation direction of the induced hydraulic fractures was north by east; therefore, hydraulic fractures intersected with natural fractures, which led to the complex fracture system and showed many cross-cutting linear features.

**Figure 2:**
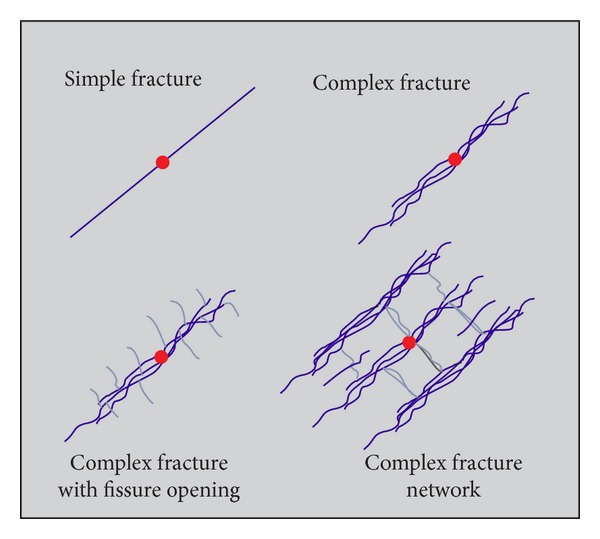
Classification of fracture from simple to complex [21].

Based on fracture extension characteristic in shale reservoirs, Warpinski et al. [21] classified hydraulic fractures into four major categories: the single plane biwing fracture, complex multiple fracture, complex multiple fracture with open natural fractures, and complex fracture network, as shown in Figure 2. Warpinski et al. also believed that complex fracture network was formed after fracturing in shale reservoirs.

## 3. Geologic Factors Affecting Fracture Network

Several important geological factors affecting the hydraulic fracture propagation include rock mineral composition, rock mechanics properties, horizontal stress field, and distribution of natural fractures.

### 3.1. Mineral Composition

Rock brittleness is, to a large degree, controlled by its mineralogy [22, 23]. As brittleness mineral concentration, including quartz, feldspar, and calcite which contains silicon or calcium, increases and the clay concentration reduces, the rock brittleness gets higher and the development of natural fractures becomes better. Then the induced fracture network is easily formed during fracturing of shale reservoirs, which is beneficial for shale gas production. However, the volume of clay mineral as a percentage of the total matrix averages more than 30% for the whole core. This amount of clay will limit the effective gas filled porosity of shale and should make the shale softer and more ductile. Then hydraulic fracture mostly extends into simple plane fracture instead of fracture network [24]. Hydraulic fracture propagation mode and shale gas exploitation showed that the rock brittleness mineral concentration was between 46% and 60% [25], which could bring economic production. The result showed that threshold condition of brittleness mineral concentration for forming a complex fracture network was 45%.

### 3.2. Rock Mechanics Properties

The concept of rock brittleness combines both Poisson's ratio and Young's Modulus. These two components are combined to reflect the rock ability to fail under stress (Poisson's Ratio) and maintain a fracture (Young's Modulus) once the rock fractures.In terms of Poisson's Ratio, the lower the value, the more brittle the rock, and as values of Young's Modulus increase, the more brittle the rock be. Rickman et al. proposed to calculate rock brittleness by Young's Modulus and Poisson's Ratio, and the following equations are used [26]:

(1) BRIT−E=((E−1)(8−1))×100,BRIT−ν=((ν−0.4)(0.15−0.4))×100,BRIT−T=(BRIT−E+BRIT−ν)2,

where *E* is the rock elastic modulus, MPa; *ν* is the rock Poisson's ratio, dimensionless; *B*_RIT−*E*_ is brittleness component corresponding to elastic modulus, dimensionless; *B*_RIT−*ν*_ is brittleness component corresponding to Poisson's ratio, dimensionless; *B*_RIT−*T*_ is the total brittle index, dimensionless.

**Figure 3:**
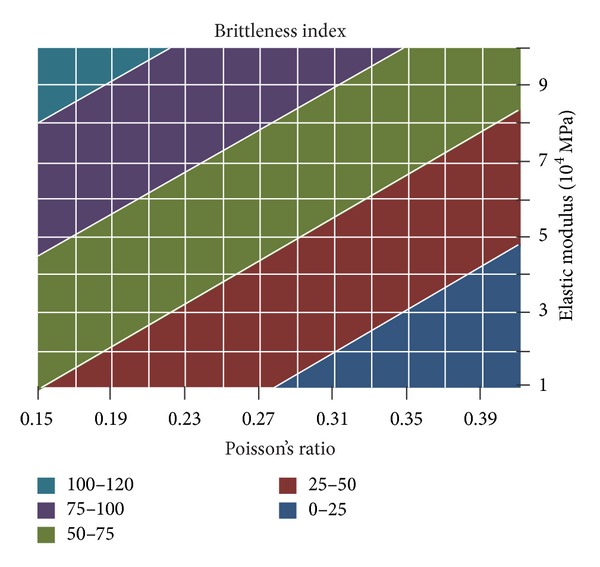
Relationship between rock brittle index and rock mechanics parameters.

According to (1), the correlation diagram of rock brittleness and rock mechanical parameters can be calculated, as shown in Figure 3. Brittleness index is the function of elastic modulus and Poisson's ratio; on the whole, rock brittleness index is higher under the condition of high elastic modulus and low Poisson's ratio.

**Figure 4:**
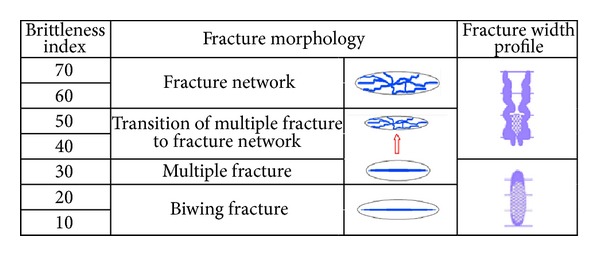
Effect of rock brittleness index on hydraulic fracture pattern [26].

Rickman et al. proposed the correlation between the rock brittleness index and fracture morphology, as shown in Figure 4. As brittleness increases, the fracture geometry becomes more complex. When the brittleness index of shale is lower, it is easy to form the conventional biwing fracture. While the brittleness index is more than 60, the fracture will extend into fracture network.

### 3.3. Distribution of Natural Fractures

**Figure 5:**
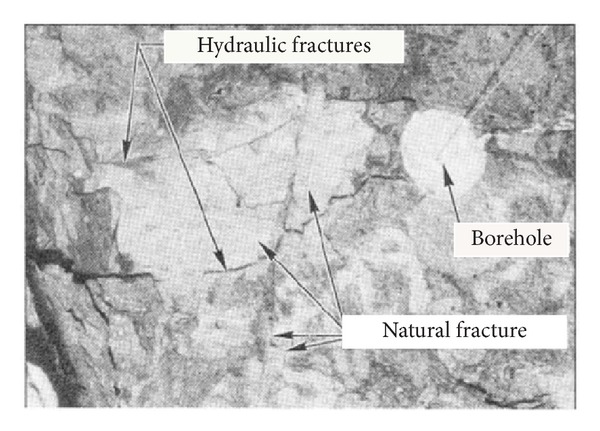
Complex fracture extension in fractured reservoir [4].

In the fracturing process of shale reservoir, natural fractures are activated to broaden the hydraulic fractures extending area and conduct gas from shale matrix to well, which is the key factor to improve the stimulation effect [27]. In fact, any hydraulic fracture extension in fractured reservoir will be influenced by natural fractures; hydraulic fracturing field test in fractured formations provided a visual observation for the complex geometry of hydraulic fractures [4], as shown in Figure 5; single fracture extension could not be observed and more complex branching fractures extending showed after fracturing.

**Figure 6:**
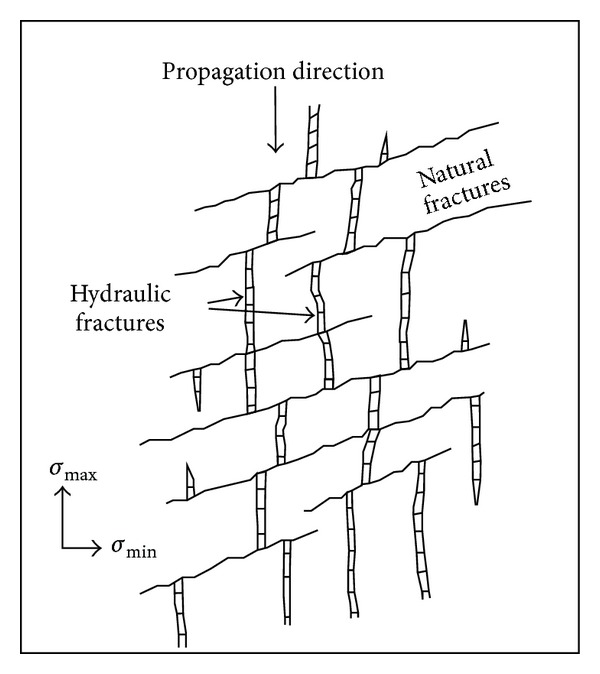
Visualization of far-field fracture network in fractured rock mass [4].

Based on the results analysis of the field test, Warpinski and Teufel believed that the geometric shape of hydraulic fractures was a fracture zone with width about 6–9 m and put forward the idea of far well fracture propagation zone [4]. As shown in Figure 6, hydraulic fracture presented multiple parallel branching extension form. At the same time, we could find the more developed natural fractures had a greater effect on hydraulic fracture extension, which made the extending mode of hydraulic fracture more complex.

### 3.4. Horizontal Stress Field

**Figure 7:**
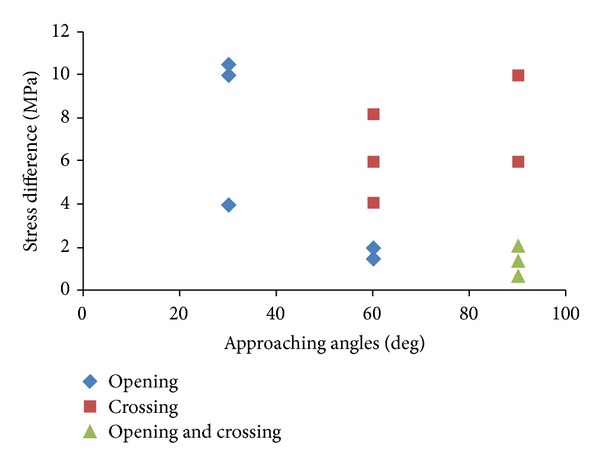
Experimental results of the impact of stress difference and approaching angles on fracture propagation [8].

According to fracture network extension mode in Figure 6, the fracture network is essentially controlled by the intersecting of hydraulic fracture and natural fractures. Blanton [7, 8] implemented the simulation experiment about hydraulic fracture propagation path when it intersected with natural fractures through triaxial stress experiment, as shown in Figure 7. The experiment results showed that hydraulic fracture would propagate along natural fractures under low horizontal stress difference condition and would cross natural fractures under high horizontal stress difference condition. However, under the high approaching angles and low horizontal stress difference, hydraulic fractures extending along and cross natural fracture would appear simultaneously.

**Figure 8:**
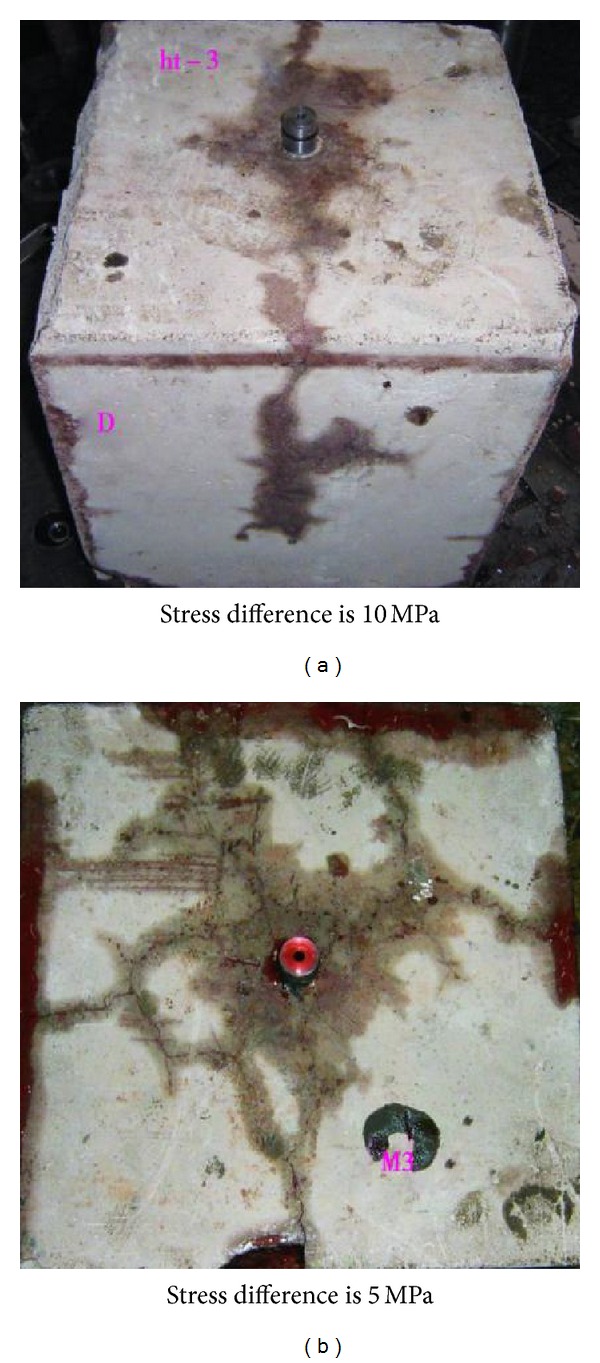
Experimental results contract of fracture propagation pattern for different stress difference [12].

Chen et al. [12] confirmed that the fracture network extending pattern is associated with horizontal principal stress difference by a large size triaxial experiment system. Under the condition of high horizontal principal stress difference, a main fracture and some small multibranch fractures would form, but under the condition of low horizontal principal stress difference, a radial fracture network would be induced, as shown in Figure 8. Hence, fractured formations with low horizontal principal stress difference possess the weak stress anisotropy, and the treating net pressure difference is less when fractures propagate along different direction. So it is easier for hydraulic fracture to propagate along natural fractures in random direction to form fracture network.

## 4. Engineering Factors Affecting Fracture Network

The engineering factors which influence fracture network extension in shale formation include three aspects: treating net pressure, fluid viscosity, and fracturing scale.

### 4.1. Treating Net Pressure

Olson and Dahi-Taleghani [28] researched the impact of net pressure on the interaction of hydraulic facture and natural fractures in the fractured reservoir by the boundary element method and proposed the concept of the net pressure coefficient as follows:

(2) $$\begin{array}{c} R_{n}=\frac{P_{\text{frac}}-\sigma_{\min}}{\sigma_{\max}-\sigma_{\min}}, \end{array}$$

where *P*_frac_ is fracture fluid pressure, MPa, and *σ*_max⁡_ and *σ*_min⁡_ are, respectively, the horizontal maximum and minimum principal stress, MPa.

**Figure 9:**
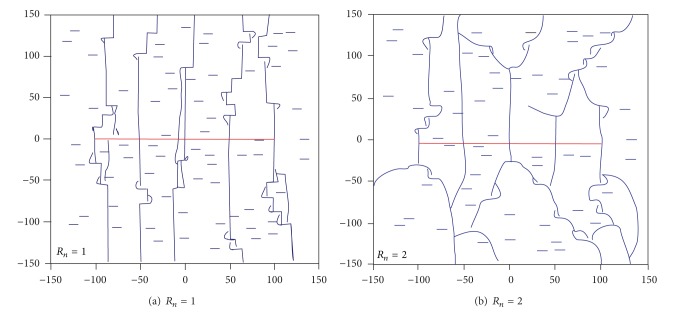
Hydraulic fracture propagation pattern for horizontal wells under different net pressure [28].

Considering the natural fractures strike along the horizontal minimum principal stress direction, fracture propagation direction would be perpendicular to the natural fractures. The simultaneous propagation of hydraulic fractures from the 5 different horizontal shooting points was simulated when *R*_*n*_ equaled to 1 and 2, respectively. The simulated results were shown in Figure 9, and, as can be seen from the contrast, the greater treating net pressure would cause more complex fracture extension.

**Figure 10:**
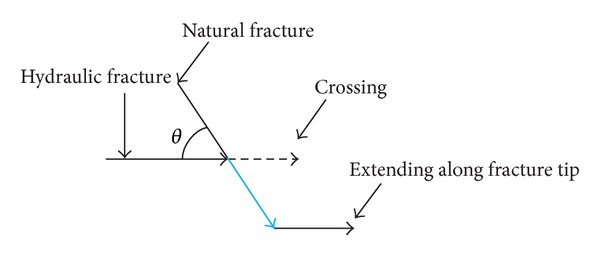
Diagram of hydraulic fracture reorienting propagation from natural fracture tip.

From Figure 6, the plane frame of intersection of hydraulic fracture and natural fracture can be obtained, as shown in Figure 10 [29]. During hydraulic fracturing, if hydraulic fracture propagates along the natural fractures tip when it intersects with natural fracture, it is possible to induce the branch of hydraulic fracture to form a complex fracture network. However, the fluid pressure of intersection point needs to overcome the fluid pressure drop from intersecting point to natural fracture tip and meet the initiation and propagation condition at natural fracture tip.

According to the theory of elasticity, the mechanics condition of fracture propagation from natural fracture tip is as the following equation [29]:

(3) $$\begin{array}{c} p_{i}\left(t\right)-\Delta p_{\text{nf}}>\sigma_{\text{n}}+T_{o}, \end{array}$$

where *σ*_n_ is the normal stress acting on the natural fracture, MPa; *T*_*o*_ is the rock tensile strength, MPa; Δ*p*_nf_ is fluid pressure drop between intersection point and natural fracture tip, MPa; *p*_*i*_(*t*) is the fluid pressure at the intersection point, MPa.

Considering that hydraulic fracture is blunted at the interface, the fluid pressure of intersection point of hydraulic fracture and natural fracture can be expressed as follows:

(4) $$\begin{array}{c} p_{i}\left(t\right)=\sigma_{\min}+p_{\text{net}}, \end{array}$$

where *p*_net_ is the treating net pressure, MPa.

The normal stress acting on the natural fracture is

(5) $$\begin{array}{c} \sigma_{\text{n}}=\frac{\sigma_{\max}+\sigma_{\min}}{2}+\frac{\sigma_{\max}-\sigma_{\min}}{2}\cos2\left(90^{\text{o}}-\theta \right), \end{array}$$

by submitting (4) and (5) into (3), the following equation can be obtained:

(6) $$\begin{array}{c} p_{\text{net}}>\frac{1}{2}\left(\sigma_{\max}-\sigma_{\min}\right)\left(1-\cos2\theta \right)+T_{o}+\Delta p_{\text{nf}} \end{array}$$

and Δ*p*_nf_ can be calculated by the following equation [30]:

(7) Δpnf=4(pi−p0)π   ×∑n=0∞12n+1exp⁡[−(2n+1)2π2knft4ϕnfμCtLnf2]sin(2n+1)π2,

where *k*_nf_ is natural fracture permeability, mD; *ϕ*_nf_ is natural fracture porosity, dimensionless; *μ* is reservoir fluid viscosity, mPa s; *C*_t_ is natural fracture comprehensive compression factor, 1/MPa; *p*_0_ is the reservoir initial fluid pressure, MPa; *p*_*i*_ is the fluid pressure at intersection point of hydraulic fracture and natural fracture, MPa; *L*_nf_ is natural fracture length, m.

**Figure 11:**
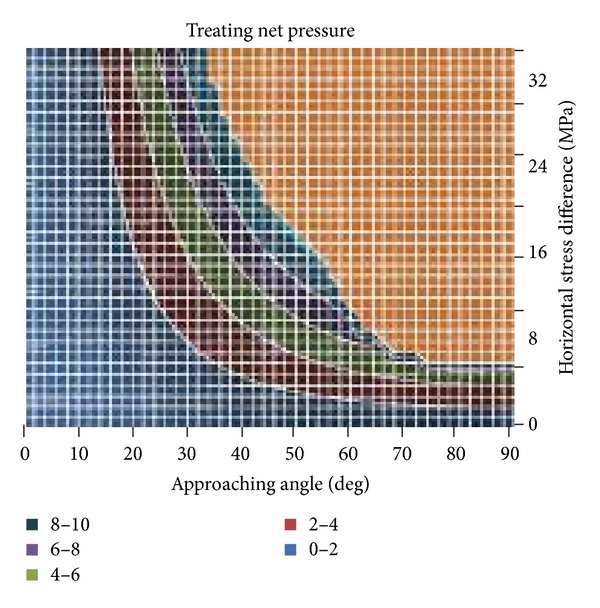
Net pressure of hydraulic fracture reorienting propagation along natural fracture for different approaching angles and horizontal stress difference.

According to (6), we can calculate the treating net pressure when hydraulic fracture propagates along natural fractures under different horizontal stress difference and different approaching angles, as shown in Figure 11. Clearly, if the horizontal stress difference and the approach angle are high, the complex fracture network can grow only if a high net pressure can be developed.

Based on the above results of numerical simulation and theoretical analysis, we can see that improving the treating net pressure is favorable of forming a complex fracture network for shale reservoir fracturing.

### 4.2. Fluid Viscosity

The fracturing fluid viscosity in shale reservoir has an important influence on the complexity of fracture extension. The fluid viscosity gets higher; the complexity of fracture extension will reduce remarkably [31]. In the section, the effect of the fracturing fluid viscosity on hydraulic fracture network propagation is analyzed through laboratory experiments and field fracturing practice.

**Figure 12:**
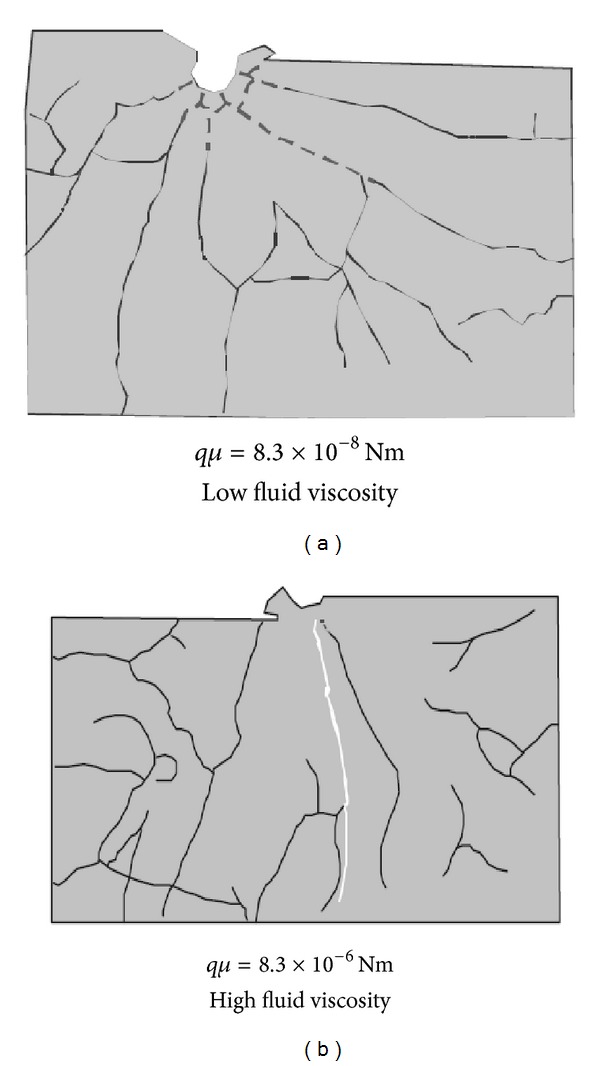
Experimental results contract of fluid viscosity impact on fracture pattern [14].

Beugelsdijk et al. [14] have studied the effect of fracturing fluid viscosity on the hydraulic fracture propagation in fractured formations by laboratory experiment, as shown in Figure 12. The experimental results showed that the operation pressure curve did not present the characteristics of fracture initiation for the injection of low viscosity fluid. By observing rock body, it was found that there was no main fracture along the horizontal maximum principal stress direction and many fractures always extended along natural fractures. However, main fracture could be obviously visible when high fluid viscosity was injected, and hydraulic fracture hardly reacted with natural fractures. From the experimental results, it can be seen that low fluid viscosity is conducive to forming complex fracture network and high fluid viscosity is good for forming a single fracture.

**Figure 13:**
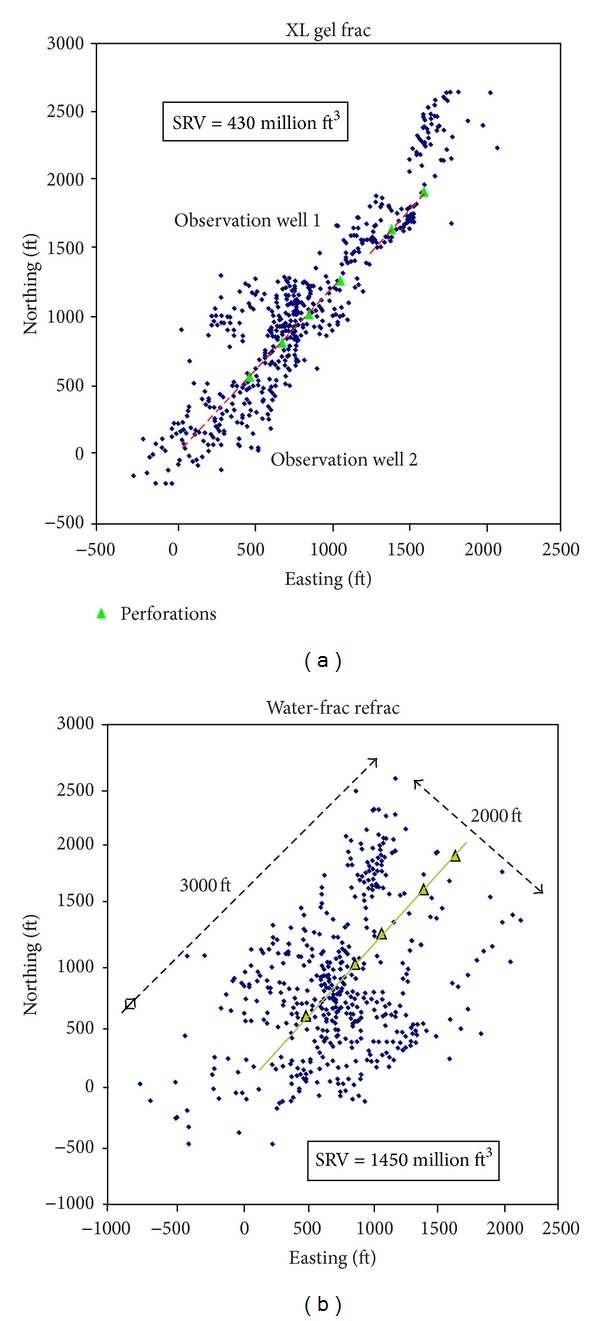
SRV comparison of using gel fracturing fluid and slickwater fracturing fluid [34].

The field treating data showed that the injection of high viscosity fluid will reduce the complexity of fracture network [32, 33]. Based on microseismic monitoring mapping of two operations with different fracturing fluid on a same horizontal well in shale [34], Cipolla et al. [35] calculated and contrasted the SRV when two kinds of fracturing fluid (gel fracturing fluid and slickwater) were, respectively, taken. As obviously shown in Figure 13, the SRV by using slickwater was much larger than the SRV by taking gel fracturing fluid, which indicated that slickwater could easily form the complex fracture network. The research result provides an important basis for shale stimulation to select low viscosity fluid preferably.

Laboratory experiment and fracturing practice analysis obviously show that fracturing fluid viscosity plays an important role in the fracture network complexity. The selection of fracturing fluid with low viscosity is more favorable for the generation of a complex fracture network. Due to lower fracturing fluid viscosity, the fluid pressure conduction is easier, and fluid pressure drop is smaller in natural fractures; hence, the fluid pressure at natural fracture tip is easier to reach the pressure threshold that initiation and propagation of hydraulic fracture needs.

### 4.3. Fracturing Scale

Traditional fracturing theory believes that as the fracturing scale gets larger, the hydraulic fracture half-length will become longer. However, for the fracturing of shale reservoir, there is the same relevance between fracturing scale and the SRV.

**Figure 14:**
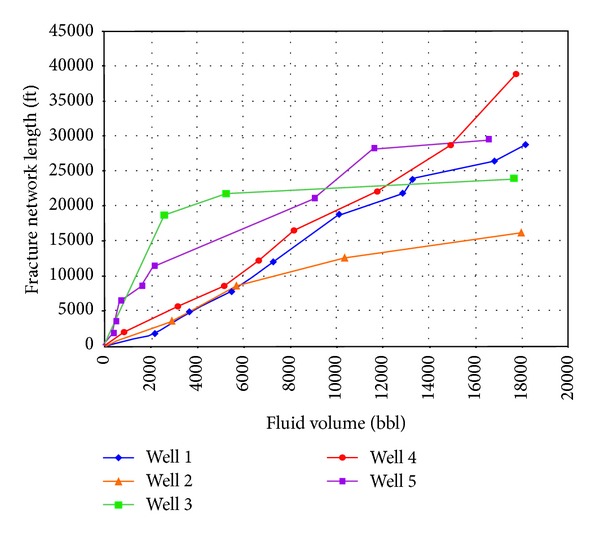
Relationship between total fracture network length and total pumped fluid volume [20].

Mayerhofer et al. [19] firstly proposed this concept of stimulated reservoir volume (SRV) in 2006 when they studied the microseismic monitoring mapping and fracture morphology variation characteristics in Barnett shale. Research results showed that the bigger the SRV is, the higher the shale well production is. Then increasing SRV was proposed to improve stimulation effect in the shale fracturing. Mayerhofer et al. [20] further discussed the relationship between fracturing scale and the total length of fracture network in Barnett shale 5 wells, as shown in Figure 14, which showed that the bigger the pumped fracturing fluid volume is, the more complex the fracture network is and the longer the total length of fracture network is.

**Figure 15:**
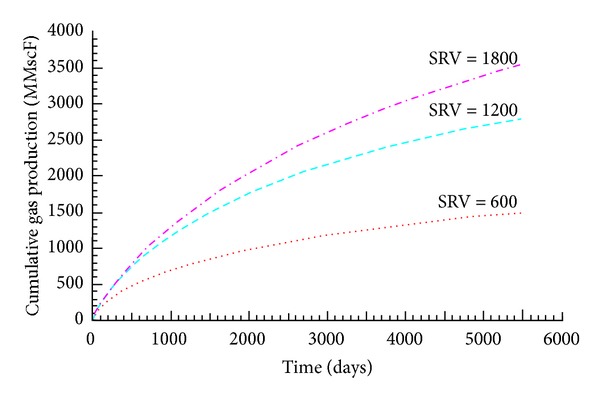
Effect of SRV on cumulative gas production for shale fracturing well [19].

Many researchers have studied the impact of shale fracturing scale on the production after fracturing [3, 19, 21]. Mayerhofer et al. [19] combined microseismic monitoring mapping and numerical simulation method to analyze the relationship between the SRV and the production after fracturing in shale reservoir, as shown in Figure 15, which showed the positive correlation relationship between the SRV and the production.

Hossain et al. [36] have proposed that well production increase was mainly from dilation of natural fracture network for naturally fractured reservoirs. Accordingly, for shale reservoirs, the greater the fracturing scale is, the more complex the fracture network propagation is, and the higher the corresponding well production is. Hence, using large fracturing scale to increase the SRV is an important measure to improve the stimulation effect and the production of the shale well.

## 5. Conclusions

This paper discusses the controlling factors of fracture extending into network in the shale reservoir from the geological and the engineering factors: according to reservoir geological attributes, higher brittle mineral contents of rock, stronger elastic characteristic of rock mechanical properties, smaller horizontal differential stress, and better developed natural fractures will be constructive to better extension and propagation of hydraulic fractures extending into network; according to engineering conditions of fracturing operations, higher treating net pressure, lower fluid viscosity, and larger fracturing scale will be more helpful to form a fully propagated fracture network.

The forming of fracture network is the key to obtain effective development in shale formation. The special hydraulic fracture propagation in shale reservoir broadens the understanding to conventional fracturing technology. Because of the above research results, this paper has an important theoretical and practical significance to understand the extending mechanism and regularity of fracture network in shale formation and can improve the science and effectiveness of fracturing design for shale reservoir.
